# Rho GTPases in human breast tumours: expression and mutation analyses and correlation with clinical parameters

**DOI:** 10.1038/sj.bjc.6600510

**Published:** 2002-09-04

**Authors:** G Fritz, C Brachetti, F Bahlmann, M Schmidt, B Kaina

**Affiliations:** Institute of Toxicology, Division of Applied Toxicology, University of Mainz, Obere Zahlbacher Str. 67, D-55131 Mainz, Germany; Department of Obstetrics and Gynecology, University of Mainz, Obere Zahlbacher Str. 67, D-55131 Mainz, Germany

**Keywords:** Rho GTPases, breast tumours, tumour progression, mutation analysis

## Abstract

In the present study, we addressed the question of a putative relevance of Rho proteins in tumour progression by analysing their expression on protein and mRNA level in breast tumours. We show that the level of RhoA, RhoB, Rac1 and Cdc42 protein is largely enhanced in all tumour samples analysed (*n*=15) as compared to normal tissues originating from the same individual. The same is true for ^32^P-ADP-ribosylation of Rho proteins which is catalysed by *Clostridium botulinum* exoenzyme C3. Also the amount of Rho-GDI and ERK2 as well as the level of overall ^32^P-GTP binding acvitity was tumour-specific elevated, yet to a lower extent than Rho proteins. Although the amount of Rho proteins was enhanced in tumours, most of them did not show changes in *rho* mRNA expression as compared to the corresponding normal tissue. Thus, elevated gene expression seems not to be the underlying mechanism of tumour-specific overexpression of Rho proteins. Sequence analysis of RhoA, RhoB, RhoC and Rac1 failed to detect any mutations in both the GTP-binding site and effector binding region. By analysing >50 tumour samples, the amount of RhoA-like proteins (i.e. RhoA, B, C), but not of Rac1, was found to significantly increase with histological grade and proliferation index. Rho protein expression was neither related to p53 nor to HER-2/neu oncogene status. Expression of *rho* mRNAs did not show a significant increase with histological grade. Overall the data show that (1) Rho proteins are overexpressed in breast tumours (2) overexpression is not regulated on the mRNA level (3) the expression level of RhoA-like proteins correlates with malignancy and (4) Rho proteins are not altered by mutation in breast tumours.

*British Journal of Cancer* (2002) **87**, 635–644. doi:10.1038/sj.bjc.6600510 www.bjcancer.com

© 2002 Cancer Research UK

## 

The Ras homologous (=Rho) subfamily of low molecular mass (*M*_r_ ∼21 kDa) GTP-binding proteins encompasses RhoA-like (e.g. RhoA, B, C), Rac and Cdc42 proteins. Among the Rho GTPases, specifically RhoA-like proteins have been shown to be ADP-ribosylated by exoenzyme C3 from *Clostridium botulinum* which leads to their inactivation (Aktories, 1997). Using this type of analysis as well as by microinjection of constitutively activated V14RhoA, the involvement of Rho proteins in the regulation of the organisation of the actin cytoskeleton has been demonstrated (Chardin *et al*, 1989; Paterson *et al*, 1990; Wiegers *et al*, 1991). Rho proteins are reported to be associated with various kinases such as protein kinase N (PKN) (Watanabe *et al*, 1996), phosphoinositide-kinase (Tolias *et al*, 1995) and Rho binding kinase (Rock) (Leung *et al*, 1995). The latter influences the actin cytoskeleton through phosphorylation of the myosin light chain (Amano *et al*, 1996).

Whereas RhoA is thought to interfere mainly with stress fibre formation, Rac and Cdc42 are believed to regulate the formation of lamellipodia and filopodia, respectively (Nobes and Hall, 1995; Hall, 1998). In addition to their interference with the microfilamental network, Rho GTPases participate in the regulation of endocytosis (Lamaze *et al*, 1996), cell cycle progression (Olson *et al*, 1995), differentiation (Fritz *et al*, 1994a), genotoxic stress-induced signalling (Coso *et al*, 1995; Hill *et al*, 1995; Minden *et al*, 1995) and notably also malignant transformation. For example, transforming potency has been reported for RhoA (Avraham and Weinberg, 1989; Perona *et al*, 1993). Both RhoA and Rac as well as RhoB are essential for Ras-mediated transformation (Khosravi-Far *et al*, 1995; Prendergast *et al*, 1995; Qiu *et al*, 1995). Furthermore, Rho GTPases interfere with cadherin dependent cell-cell contacts as well as with integrin function (Laudanna *et al*, 1996; Schwarz *et al*, 1996; Braga *et al*, 1997). In addition, guanine exchange factors (GEFs) for Rho GTPases such as Dbl and Tiam exhibit oncogenic potency (Habets *et al*, 1994; Yaku *et al*, 1994; Michiels *et al*, 1995; van Leeuwen *et al*, 1995; Schwartz *et al*, 1996). As analysed *in vivo* by a mouse xenograft model system, it has been suggested that RhoA and RhoC play a central role in the process of invasion and metastasis (Imamura *et al*, 2000; del Peso *et al*, 1997; Itoh *et al*, 1999; Yoshioka *et al*, 1999; Clark *et al*, 2000). As demonstrated by a chip-based method, RhoC is involved in mediating metastasis of both murine and human melanoma cell lines (Clark *et al*, 2000). Altogether, data available are indicative of a possible role of Rho GTPases in tumour development and progression (Price and Collard, 2001; Schmitz *et al*, 2000). Yet, studies on human tissues supporting this hypothesis are largely missing. It is well established that overexpression and activation of oncogenes or inactivation of tumour suppressor genes are related to tumour formation, and often they are used as prognostic markers (Bos, 1988; Anderson *et al*, 1992; Knudson, 1993). In particular, point mutations or amplification of members of the *ras* gene family have been found in a variety of human tumours (Bos, 1988). However, only very few reports are available so far dealing with the analysis of Rho expression or Rho mutation in human tumours (Suwa *et al*, 1998a; Fritz *et al*, 1999b; Schnelzer *et al*, 2000).

Thus, despite promising *in vitro* and animal studies (Schmitz *et al*, 2000; Price and Collard, 2001), convincing evidence for the involvement of Rho GTPases in human carcinogenesis in particular in tumour progression and invasiveness is largely missing. In the present study we analysed the expression of different types of Rho GTPases in breast tumours, both on the level of the protein and mRNA. In order to take into account a possible interindividual variation in the expression level of Rho, we compared tumorigenic tissue with the corresponding normal tissue originating from the same patient. We also addressed the question of a putative correlation of particular Rho species with established prognostic breast tumour markers and investigated whether or not regulatory domains of Rho proteins are affected by mutations in tumours.

## MATERIALS AND METHODS

### Materials

Normal and tumorigenic tissues from breast used in the present study were subjected to pathological consideration (as to histological grade, proliferation index (MIB-1), p53 and HER-2/neu status) before they were used for biochemical analyses. RhoA (number sc-179, rabbit polyclonal), RhoB (number sc-180, rabbit polyclonal) and ERK2 (number sc-154, rabbit polyclonal) specific antibodies were purchased from Santa Cruz (San Diego, USA), Rho antibody detecting all RhoA-like species (i.e. RhoA, B and C) was purchased from BD Transduction Laboratories (number R73920, mouse monoclonal) (Lexington, USA). The same is true for Rac1 (number R56220, mouse monoclonal), Cdc42 (number C70820, mouse monoclonal) and Rho GDI (number R26320, mouse monoclonal) antibodies. Clostridium botulinum exoenzme C3 was generously provided by I Just (Hannover, Germany).

### Preparation of tissue extracts

Frozen normal and tumorigenic tissues originating from the same patient were dissected with a microtome. Extraction of proteins from cut slices was done as described (Fritz *et al*, 1999b). Soluble fraction was obtained by centrifugation (10 000 **g**, 4°C, 10 min). Protein determination was performed according to Bradford (Bradford, 1976). Extracts were frozen in liquid nitrogen and stored at −80°C.

### ADP-ribosylation assay

^32^P-ADP-ribosylation with 25 μg of protein from cytosolic extracts was performed as described (Fritz *et al*, 1994b). Reaction products were separated by 12.5% SDS–PAGE. After Coomassie staining gels were dried and subjected to autoradiography. For quantitation, densitometrical analysis was performed. For calculation of relative level of ADP-ribosylation of tumours, ADP-ribosylation of extracts from the human breast carcinoma cell line MCF-7 (grown in DMEM+10% FCS) was set to 1.0.

### Western blot analysis

For immunological detection of Rho proteins 30–50 μg of cytosolic proteins were separated by SDS–PAGE (12.5% gel). After wet-blotting to nitrocellulose, proteins bound to the membrane were stained with Ponceau S in order to confirm that identical amounts of protein have been transferred. Expression of Rho GTPases (i.e. RhoA, RhoB, Rac, Cdc42) and the Rho-regulatory protein Rho GDI was analysed using the corresponding Rho specific antibodies (Santa Cruz and BD Transduction Laboratories). For determination of RhoA-like proteins an antibody cross-reacting with RhoA, B and C was used (number R73920) (BD Transduction Laboratories). As confirmed by comparative analysis of a panel of tumour samples this antibody showed identical expression pattern as a RhoA specific one (number sc-179) (Santa Cruz). This finding supports previous observation that RhoA is the quantitatively predominant Rho GTPase within the family of RhoA-like GTPases (Fritz *et al*, 1995). As described previously, RhoA specific antibody (number sc-179; Santa Cruz) does not cross-hybridise with RhoB protein and the other way around (Fritz *et al*, 1999a). After incubation with peroxidase coupled anti-rabbit IgG and anti-mouse IgG, respectively, proteins were visualised by chemiluminescence. Filters were repeatedly reprobed, whereby finally, as a loading control, ERK2 specific antibody was used. Relative Rho protein levels were calculated by referring them to the amount of ERK2 protein. As a further control, the expression of Rho proteins in extract from human breast carcinoma cell line MCF-7 was determined. Autoradiograms were quantified by densitometry (software: Bio Image IQ).

### GTP-overlay assay

Membrane proteins (10 000 **g** pellet fraction) were separated by SDP–PAGE (15% gel) and blotted to nitrocellulose. Afterwards, proteins were renatured by overnight incubation in buffer containing 25 mM Tris/192 mM glycine. After 20 min of preincubation in binding buffer (50 mM Tris (pH 7.5), 0.3% Tween 20, 5 mM MgCl_2_, 1 mM EGTA), α-^32^P-GTP (1 μCi ml^−1^) was added. After a further incubation period of 90 min at room temperature, filters were washed three times for 30 min with binding buffer. Subsequently, the level of ^32^P-GTP binding was visualised by autoradiography. As a control, GTP-binding capacity of extracts from MCF-7 cells was determined. Binding activity of tissue extracts was related to that of MCF-7 cell extract which was set to 1.0. This assay aimed at examining whether tumorigenic tissue differs from normal tissue with respect to the overall expression of GTP binding proteins, not at analysing differences in the expression level of a single GTPase species.

### Analysis of *rho* mRNA expression

In order to analyse the expression of the diverse Rho species on the level of the mRNA, total RNA was isolated from breast tissue samples using the Quiagen RNA extraction kit (Quiagen, Hilden, Germany). One μg of RNA was used for semiquantitative RT–PCR analysis (Titan One tube PCR kit, Roche Diagnostics GmbH). The sequence of the primer pairs for specific amplification of *rhoA, rhoB, rhoC, rac1*, Ki-Ras and GDH were the following: *rhoA*, (582 bp PCR product): (1) 5′-ATGGCTGCCATCCGGAAGAAA-3′ and (2) 5′-TCACAAGACAAGGCAACCAGA-3′; *rhoB*, (548 bp PCR product): (1) 5′-GCGTGCGGCAAGACGTCTG-3′ and (2) 5′-TCATAGCACCTTGCAGCAGTT-3′; *rhoC*, (582 bp PCR product): (1) 5′-ATGGCTGCAATCCGAAAGAAG-3′ and (2) 5′-TCAGAGAATGGGACAGCCCCT-3′; *rac1*, (448 bp PCR product): (1) 5′-CATCAAGTGTGTGGTGGTGGG-3′ and (2) 5′-TTACAGCACCAATCTCCTTAG-3′; Ki-Ras, (405 bp PCR product): (1) 5′-AGCCTGTTTTGTGTCTACTGTT-3′ and (2) 5′-GAGAGGCCTGCTGAAAATG-3′; GDH, (392 bp PCR product): (1) 5′-GTCTTCACCACCATGGAGAAGGCT-3′ and (2) 5′-CATGCCAGTGAGCTTCCCGTTCA-3′. For specific amplification of *rac1* splice variant *rac1b* (Jordan *et al*, 1999; Schnelzer *et al*, 2000) the following two sets of primer pairs were used (1) 5′-CATCAAGTGTGTGGTGGTGGG-3′ and (2) 5′-GGCAATCGGCTTGTCTTTGCC-3′ resulting in PCR product of 274 bp as well as (1) 5′-GGAGAAACGTACGGTAAGGAT-3′ and (2) 5′-TTACAGCACCAATCTCCTTAG-3′ leading to an amplification product of 287 bp in length. Rac1b mRNA expression was analysed by nested PCR of the *rac1* amplification reaction. For PCR amplification 30 cycles were performed (annealing condition: 55°C, 2 min). PCR products were separated onto 1.5% agarose gels and visulised by ethidium bromide staining. Quantitation of PCR products was performed by use of image analysis software (Multi-analyst; Bio-Rad Laboratories, CA, USA). Specificity of *rho* amplification products (i.e. *rhoA, rhoB, rhoC*) was confirmed by diagnostic restriction enzyme digests using enzymes specifically cutting a particular rho cDNA species. Relative *rho* mRNA expression was calculated by referring *rho* mRNA level to that of GDH or Ki-Ras mRNA level.

### Sequence analysis

To investigate whether or not mutations of Rho GTPases do occur in human breast tumours, PCR products were subjected to automated sequencing (373A DNA Sequencer from ABI). To detect putative mutational changes in central regulatory domains of Rho proteins, we focused on sequencing nucleotides 1-285 (AA 1-95), covering the GTP-binding and effector binding domains of Rho GTPases.

## RESULTS

**Figure 1:**
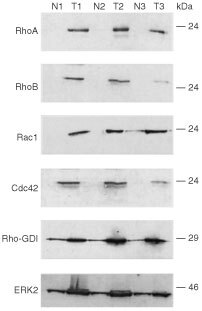
Expression of Rho GTPases in malignant and non-malignant breast tissue. Each 50 μg of protein isolated from tumorigenic (T1, T2, T3) and corresponding normal breast tissue (N1, N2, N3) originating from the same patient was separated by SDS–PAGE and subjected to Western blot analysis using the antibodies indicated. Shown is the autoradiography.

To address the question of putative relevance of Rho proteins in human carcinogenesis and tumour progression, we compared the expression of Rho GTPases in tumours from breast with that of normal tissue originating from the same individual. As representatively shown in Figure 1A

for three pairs of non-malignant *vs* corresponding malignant tissues, RhoA, RhoB, Rac1 and Cdc42 are overexpressed on protein level in breast tumours. In normal tissue, the expression of these Rho proteins is hardly or even not detectable. The latter is true in particular for RhoA and RhoB. Because RhoC specific antibody is not available, we were unable to analyse the expression of this Rho species on the level of the protein. In contrast to Rho GTPases, the expression of the Rho regulatory factor Rho-GDI is easily detectable both in tumour and in the normal tissue. The same is true for ERK2. In Table 1Table 1

**Table 1:**
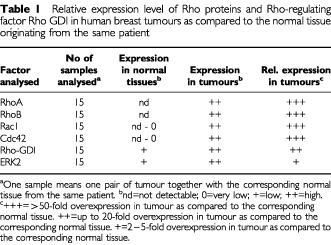
Relative expression level of Rho proteins and Rho-regulating factor Rho GDI in human breast tumours as compared to the normal tissue originating from the same patient

, relative levels of expression of Rho proteins, Rho-GDI and ERK2 in 15 pairs of normal *vs* malignant tissue from the same patient are compiled. In addition to Western blot analysis, we examined the expression of Rho proteins by means of *Clostridium botulinum* C3-mediated ^32^P-ADP-ribosylation (Rubin *et al*, 1988; Aktories, 1997). In line with the Western blot data, extracts from breast tumours showed largely enhanced levels of ADP-ribosylation as compared with the normal tissues, which displayed only very faint amounts of ADP-ribosylated Rho proteins (Figure 2A

**Figure 2:**
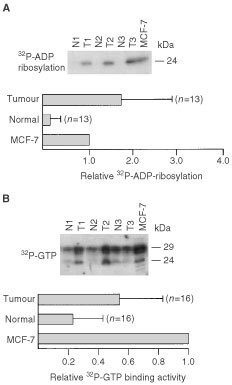
Comparative analysis of C3-mediated ^32^P-ADP-ribosylation and ^32^P-GTP binding activity of Rho proteins in human breast tumours as compared to normal tissue. (**A**) ^32^P-ADP-ribosylation of Rho proteins in extracts from tumour and corresponding non-malignant tissue. Twenty-five μg of cytosolic protein was ^32^P-ADP-ribosylated by use of exoenzyme C3 from *C. botulinum* as described in Materials and Methods. Reaction products were separated by SDS–PAGE. Autoradiography was densitometrically analysed and relative ^32^P-ADP-ribosylation of tissue extracts was related to that of MCF-7 cells, which was set to 1.0. Autoradiogram of a representative analysis of three sampled pairs is shown in the upper part of the figure. N, normal tissue; T, tumour. (**B**) Analysis of ^32^P-GTP binding activity of extracts from non-malignant and tumorigenic breast tissue. Thirty μg of protein was separated by SDS–PAGE (15% gels). After wet blotting to nitrocellulose and renaturation, ^32^P-GTP binding activity was analysed as described in Materials and Methods. Relative GTP binding activity of extracts from MCF-7 cells was set to 1.0. The autoradiogram of a representative analysis is shown in the upper part of the figure. N, normal tissue; T, tumour.

). Since Rho GTPases belong to the family of Ras-related small GTP binding proteins, we wished to know whether small GTPases in general are overexpressed in tumours and whether this happens to a similar extent as for Rho proteins. To this end we analysed the overall GTP binding activity of normal and tumorigenic tissue by performing ^32^P-GTP overlay assays. These experiments revealed that the overall GTP binding activity, representing the expression level of the family of small GTPases as a whole, was enhanced in the tumours (Figure 2B). However, it is important to note that also in the non-malignant tissue ^32^P-GTP binding activity was clearly detectable. This is in contrast to Rho proteins. Overall, the data show that Rho GTPases are highly overexpressed on the level of the protein in breast tumours. Rho-GDI, ERK2 and the overall expression of ^32^P-GTP binding proteins is also enhanced in tumours, yet to a clearly lower level than Rho proteins are. Obviously, there is a preferential overexpression of Rho GTPases in breast tumours.

**Figure 3:**
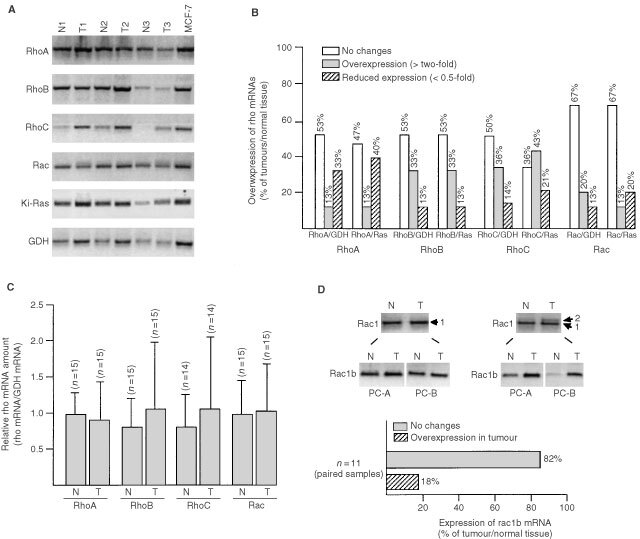
*rho* mRNA expression in tumorigenic and normal breast tissue. (**A**) Total RNA from tumorigenic and corresponding normal tissue from breast was analysed as to the expression of various *rho* mRNA species (i.e. *rhoA, rhoB, rhoC, rac*1) by RT–PCR analysis as described in Materials and Methods. As internal control expression of Ki-ras and GDH mRNA was determined. N, normal tissue; T, corresponding tumour. (**B**) Quantitative densitometrical analysis of relative *rho* mRNA expression in breast tumours *vs* normal tissue. Shown is the percentage of tumours showing similar, increased or reduced level of *rho* mRNA expression as compared to the corresponding normal tissue of the same individual. Relative expression of *rho* mRNA species was calculated by referring *rho* mRNA amount to that of either Ki-Ras (Ras) or GDH (GDH). Both types of internal reference markers gave the same results. (**C**) Interindividual variation in the relative expression of *rho* mRNAs (*rho* mRNA/GDH mRNA) in normal and tumorigenic tissues from breast. (**D**) The upper part of the figure shows the results of representative RT–PCR analyses of two different paired samples (normal tissue (N) *vs* tumorigenic tissue (T)) where *rac1b* is clearly detectable (right part) or not (left part) as a co-amplification product of the *rac1* RT–PCR reaction (Rac1). 1,2, indicates the position of *rac1* and *rac1b* amplification product, respectively. For more specific and sensitive analysis of *rac1b* expression (Rac1b), nested PCR was performed using two different types of *rac1b* specific primer combinations as described in Materials and Methods (PC-A and PC-B). In the lower part of the figure the quantitative evaluation of the expression of *rac1b* in normal breast tissue *vs* the corresponding tumorigenic tissue from the same patient (*n*=11; paired samples) is shown. Please note that the samples representatively shown under A and D are different from each other.

The high level of expression of Rho proteins in tumours raises the question as to the underlying mechanism. One possibility is that *rho* gene expression is elevated in tumours. Therefore, we investigated *rho* mRNA expression in normal tissues and tumours by use of semiquantitative RT–PCR analyses. Relative *rho* mRNA amounts were calculated by relating them to the level of GDH or Ki-ras mRNA. Surprisingly, the tremendous overexpression of Rho in tumours on the protein level (see Figure 1A) was not reflected at all on the level of the RNA (Figure 3A

). We would like to note that the data representatively shown in Figure 3A and Figure 1A are obtained from the analysis of identical samples. As shown in Figure 3B, *rho* mRNA level in tumours was in most cases not different from that of the corresponding non-malignant tissue. As referred to either Ki-ras or GDH mRNA levels, about 50–70% of the tumours showed a similar *rho* mRNA expression as the normal counterpart of the same indiviual. Ten to twenty per cent of the tumours even showed a >50% reduction in *rho* mRNA level (i.e. *rhoA* and *rac1*). Only for *rhoB* and *rhoC*, about 30% of the tumours exhibited increase in mRNA levels (Figure 3B). Overall, the data clearly demonstrate that the highly elevated expression of Rho proteins in breast tumours is not due to an increase in *rho* gene expression. The interindividual variability in *rho* mRNA expression was rather large, both in normal and malignant tissues (∼4–10-fold) (Figure 3C). With respect to *rac1* a splice variant named *rac1b* was recently cloned both from colorectal and breast tumours (Jordan *et al*, 1999; Schnelzer *et al*, 2000). Therefore we additionally investigated mRNA expression of *rac1b* in normal *vs* malignant breast tissue. Rac1b contains a 57 bp insert as compared to *rac1* (Jordan *et al*, 1999; Schnelzer *et al*, 2000). Since the *rac1* primers used span the region of the *rac1b* extra exon, *rac1b* is co-amplified by the primer combination we have used for *rac1* expression analysis, and therefore *rac1b* is occasionally detectable as a second slower migrating band (Figure 3D, right upper part). To enable a more sensitive detection of *rac1b*, we performed nested PCR reactions using two different sets of *rac1b* specific primer pairs (PC-A and PC-B) which result in *rac1b* specific amplification products of 274 and 287 bp in length, respectively (Figure 3D). Comparing normal breast tissue samples with the corresponding tumorigenic tissues originating from the same individuals (*n*=11) as to expression level of *rac1b* mRNA, we found that in most cases (i.e. 80%) *rac1b* is expressed at a very similar level in malignant and non-malignant tissue (Figure 3D, lower part). Only two of the tumours investigated showed an enhanced expression of *rac1b* mRNA as compared to their normal counterpart (Figure 3D).

**Figure 4:**
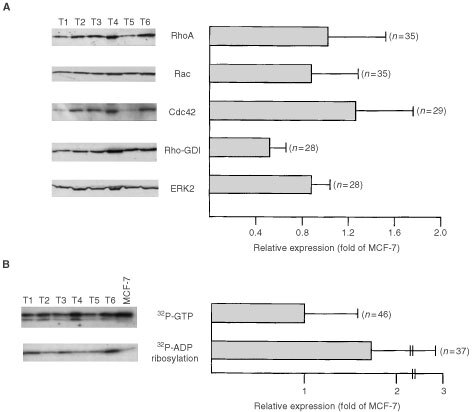
Analysis of expression of Rho proteins, ADP-ribosylated proteins and GTP-binding proteins in breast tumour samples. (**A**) Expression of Rho proteins (RhoA-like proteins (Rho), Rac1 and Cdc42) and Rho-regulatory factor Rho-GDI in breast tumours was analysed by Western blot analysis. Additionally the amount of ERK2 protein was determined. For statistical analysis, expression of Rho and Rho-GDI proteins in tumours was related to that of MCF-7 cells which was set to 1.0. In the left part of the figure an autoradiogram of a representative analysis of six tumour samples is shown. Rho, the Rho antibody used cross-reacts with the RhoA-like Rho GTPases RhoA, B and C. (**B**) Variability in ^32^P-GTP-binding activity and ^32^P-ADP-ribosylation of extracts from breast tumours. For statistical analysis, GTP-binding activity and level of ^32^P-ADP-ribosylation of breast tissue extracts was related to that of MCF-7 cells which was set to 1.0. In the left part of the figure, an autoradiography of a representative analysis of six tumour samples is shown.

**Figure 5:**
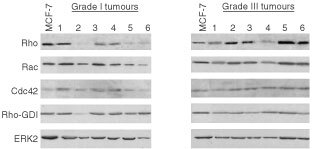
Comparative expression of Rho GTPases in breast tumours of different histological grade. Six representative samples of human breast tumours of grade I and grade III, respectively, were analysed by Western blot analysis as to the expression of various Rho GTPase, the Rho-regulatory factor Rho-GDI and ERK2. As a control, extracts from MCF-7 cells were included.

**Figure 6:**
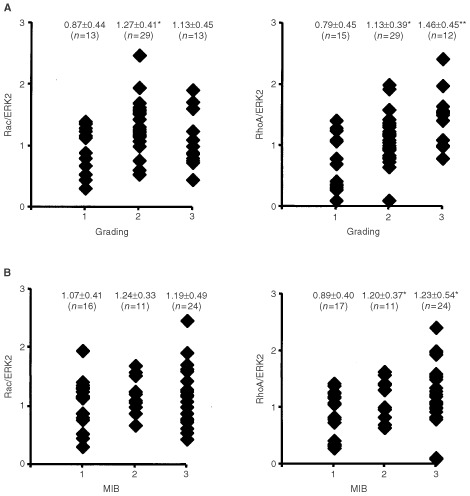
Rho protein expression correlates with histological grade and proliferation index of breast tumours. (**A**) Relative expression of RhoA-like and Rac1 proteins of breast tumours was calculated by referring the Rho protein amount to the level of ERK2 protein (RhoA/ERK2 and Rac/ERK2, respectively). Relative Rho protein expression was related to the histological grade of breast tumours. 1, 2 and 3; grade I, grade II and grade III.*, statistical significance as referred to grade I (*P<0.05; **P<0.001). (**B**) Relative RhoA and Rac1 protein expression (RhoA/ERK2; Rac/ERK2) of breast tumours was related to proliferation index (MIB-1) of the tumours. 1, 2, 3: <5%, <20%, >20% MIB-1 positive cells.*, statistical significance as referred to MIB-1 <5% (*P<0.05).

**Figure 7:**
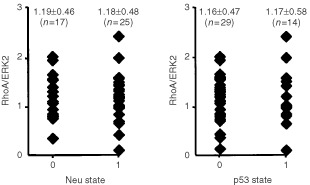
Rho protein expression is not related to HER-2/neu oncogene or p53 status of breast tumours. Relative expression of RhoA-like Rho GTPases was related to HER-2/neu oncogene and p53 status of the tumours. 0, negative for either HER-2/neu overexpression or p53 mutation; 1, positive for HER-2/neu overexpression or p53 mutation.

On the level of the protein, the interindividual variability of RhoA, Rac and Cdc42 protein expression in the tumour samples was about 3–4-fold (Figure 4A

). Variability in the expression of Rho-GDI and ERK2 proteins was much lower (<two-fold) (Figure 4A). Measuring ^32^P-GTP binding activity and the extent of ^32^P-ADP ribosylation in breast tumours, an interindividual variability of ∼3- and 10-fold, respectively, was observed (Figure 4B). To examine whether the variations in the expression level of Rho proteins in tumours might be related to histological grade, which is a generally used prognostic clinical parameter, we analysed the expression of RhoA-like GTPases (using an antibody cross-reacting with RhoA, B and C), Rac1, Cdc42, Rho-GDI and ERK2 in each of the six tumours classified as WHO grade I and grade III, respectively. As shown in Figure 5

, in particular the amount of RhoA-like protein, but not the expression of Rac1, Cdc42 or Rho-GDI seems to vary with histological grading (Figure 5). For example, as compared with MCF-7 cells, grade I tumours tended to show a reduced expression of RhoA-like proteins whereas their expression was similar or even enhanced in grade III tumours. In contrast, expression of Rac1 Cdc42, Rho-GDI and ERK2 in both grade I and grade III tumours was similar to that of MCF-7 cells (Figure 5). This initial observation indicates that among the family of Rho GTPases especially the expression of RhoA-like proteins might be related to the malignancy of the tumours. To further investigate this, we studied whether the expression of RhoA-like proteins is related to histological and prognostic clinical parameters, including grading (grade I, II and III), proliferation index (MIB) as well as HER-2/neu oncogene and p53 status. For control we included Rac1 in these studies. As an internal reference for quantitation of the amount of Rho proteins we used ERK2, because this protein showed a rather low variability in tumours (see Figure 4A). Extensive analysis of more than 50 tumours revealed that only the relative level of RhoA-like proteins (Rho/ERK2) significantly increases with histological grading from grade I to grade II (*P*<0.05) up to grade III (*P*<0.001) (Figure 6A

). In the case of Rac1, a significant increase in protein amount was only observed from grade I to grade II progression (*P*<0.05) but not to grade III. Cdc42 expression level was not significantly different between grade I and grade III tumours (data not shown). Similar data were obtained on the basis of the proliferation index of tumours as determined by quantitation of MIB (Ki-67) positive cells (Figure 6B). Whereas Rac1 protein level was not significantly related to proliferation, highly proliferative tumours (i.e. >20% MIB-1 positive cells) showed a significant increase in the level of RhoA-like proteins as compared to tumours with low proliferation index (i.e. MIB-1 <5%) (*P*<0.05). The relative amount of RhoA-like proteins was neither related to HER-2/neu oncogene expression nor p53 mutation (Figure 7

). The same is true for Rac1 proteins (data not shown). Furthermore, the expression level of RhoA was not related to the hormone (i.e. oestrogen and progesteron) status of the tumours (data not shown).

**Figure 8:**
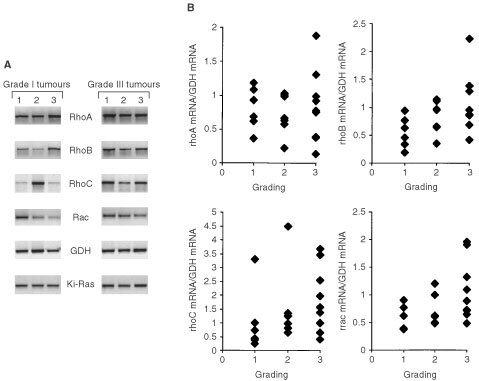
Relationship between *rho* mRNA expression and histological grade of breast tumours. (**A**) Representative analysis of *rho* mRNA expression (i.e. *rhoA, rhoB, rhoC, rac1*) in breast tumours of WHO grade I and III, respectively. As internal control mRNA expression of GDH and Ki-Ras was determined. (**B**) Correlation analysis of *rho* mRNA expression and grading status of breast tumours. Relative *rho* mRNA expression was determined by referring *rho* mRNA levels to that of GDH mRNA. 1,2,3; grade I, II and III, respectively.

Since there was a particularly large variability in the expression of *rhoB* and *rhoC* mRNAs in tumours (see Figure 3C), and having in mind a previous report indicating that *rhoC* mRNA expression is related to malignancy of pancreas tumours (Suwa *et al*, 1998a), we also examined whether *rhoB* and r*hoC* mRNA expression is related to histological grading. Initial experiments indicated that both *rhoB* and *rhoC* mRNA expression as well as *rac1* expression might vary between GI and GIII tumours, but not the expression of r*hoA* (Figure 8A

). Yet, although the expression of *rhoB*, *rhoC and rac1* tended to increase with grading (Figure 8B), the observed differences were statistically not significant. Furthermore, analysing 21 tumour samples as to the expression of the *rac1* splice variant *rac1b*, we found that also *rac1b* mRNA is expressed at similar levels in all the tumours investigated (data not shown).

Ras proteins are frequently mutated in a variety of human tumours. Therefore, in order to obtain data on the relevance of the Ras-homologous GTPases in the formation and progression of breast carcinomas, we analysed Rho proteins as to mutational alterations in tumours. These analyses were performed by sequencing the *rho* RT–PCR products. We focused on sequencing nucleotides coding for amino acids 1–95, because within this region the GTP-binding and effector binding domain of Rho GTPases is localised. Overall, 9–11 tumours and three normal tissues were subjected to sequence analysis. Neither constitutive mutational activation of RhoA, B, C or Rac1 due to V14 mutation (and V12 for Rac1, respectively), nor any amino acid exchange in their effector binding domain was detectable (Table 2Table 2

**Table 2:**
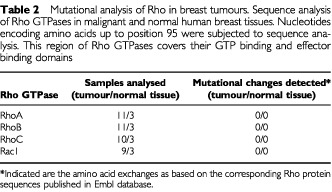
Mutational analysis of Rho in breast tumours. Sequence analysis of Rho GTPases in malignant and normal human breast tissues. Nucleotides encoding amino acids up to position 95 were subjected to sequence analysis. This region of Rho GTPases covers their GTP binding and effector binding domains

). Obviously, the regulatory domains of RhoA-like proteins and Rac1 are highly conserved and functional still intact in the malignant breast tissue.

## DISCUSSION

Although Rho GTPases have been shown to be required for cellular functions associated with tumour progression and invasiveness (Schmitz *et al*, 2000; Price and Collard, 2001), studies supporting the role of Rho for carcinogenesis and tumour progression in patients with cancer are largely missing. In the present study we aimed at (1) analysing the expression of different members of the family of Rho GTPases in breast tumours, both on the level of the protein and the mRNA, (2) establishing whether a correlation between Rho expression and clinically established diagnostic and prognostic parameters does exist and, (3) investigating whether or not Rho GTPases are mutationally altered in tumours. The data show that RhoA, Rac1 and Cdc42 proteins as well as the level of ^32^P-ADP-ribosylation are largely enhanced in malignant tissue from breast as compared with normal tissue originating from the same patient. Also the amount of RhoB, which has recently been described to interfere with cytostatic drug resistance by influencing apoptotic cell death (Fritz and Kaina, 2000; Liu *et al*, 2001), is enhanced in tumours. Moreover, the level of overall ^32^P-GTP binding activity as well as the amount of Rho-GDI protein is elevated in tumorigenic tissue, notably yet to a much lower extent than Rho proteins. In line with a previous report we also observed the level of ERK2 protein to be elevated in carcinomas as compared to the normal tissue (Sivaraman *et al*, 1997). As we have shown here, within the group of tumours, ERK2 seems to be equally distributed. Since the non-malignant tissue also displayed clearly detectable amounts of Rho-GDI, ERK2 and GTP binding activity, but no or only marginal expression of Rho proteins, the data indicate that breast tumours differ from the corresponding normal tissue notably in the overexpression of Rho GTPases.

The finding of a large overexpression of Rho proteins in breast tumours raises the question as to the underlying mechanism, in particular whether this phenomena is due to increase in *rho* gene expression. Surprisingly, in most cases (50–70%) tumorigenic and corresponding normal tissue expressed very similar levels of *rho* mRNAs (i.e. *rhoA, rhoB, rhoC, rac1*). As compared to the normal tissue, about 10–30% of the tumours revealed either enhanced levels of *rhoB* and *rhoC* mRNA or even reduced levels of *rhoA* and *rac1* mRNA. Recently *rac1* mRNA expression was reported to be enhanced in malignant *vs* benign breast tissues (Schnelzer *et al*, 2000). One possible explanation for this discrepancy might be that, in contrast to the study mentioned, we have analysed paired tissue samples, i.e. non-malignant and tumorigenic tissue originating from the same individual. Furthermore, the tumour specific increase in *rac1* mRNA expression reported in this study was rather low (i.e. ∼50%) (Schnelzer *et al*, 2000) and does not reflect the large increase in Rac1 protein expression observed in the tumours. This fact again is in line with our own data. In addition, we show that expression of the *rac1* splice variant *rac1b*, which was shown to be enhanced in colorectal tumours (Jordan *et al*, 1999), is not tumour-specific increased in breast tissue. Regarding *rac1b*, our data corroborate the study of Schnelzer *et al* (2000). Altogether our results strongly indicate that the drastic differences in Rho protein levels between malignant and non-malignant breast tissue are not due to changes in expression of the corresponding *rho* mRNAs. Thus, obviously, differences in gene expression do not account for the observed tumour specific increase in Rho proteins. We also analysed the stability of Rho proteins in the breast carcinoma cell line MCF-7 upon inhibition of protein synthesis by cycloheximide/anisomycin. These experiments showed no significant changes in Rho protein level up to 10 h after blockage of protein synthesis (data not shown). This indicates that Rho proteins are highly stable, undergoing a very slow turnover. Therefore, although we can not completely rule out the possibility that Rho proteins are rapidly degraded under *in vivo* conditions in normal breast tissue but not in tumours, it appears more likely that differences in protein stability are not responsible for the elevated level of Rho GTPases observed in breast tumours. We hypothesise that translational control mechanisms are involved in the upregulation of the amount of Rho protein in tumours.

The data also revealed broad interindividual variations in the expression of *rho* mRNAs both in normal and tumorigenic tissues. The same is true for Rho protein expression in tumours (in normal tissue Rho protein level was in general below the detection limit). In contrast to Rho GTPases, the interindividual variability in the expression of Rho-GDI and especially ERK2 protein was very low in the tumour fraction. That is why we used ERK2 protein as internal reference for calculating the relative amount of Rho protein (i.e. Rho protein level/ERK2 protein level). Correlating relative Rho protein expression with various clinical parameters we found that the level of RhoA-like GTPases significantly increases with histological grade as well as with the proliferation index (MIB-1) of the tumours. Interestingly, the expression of Rac1, which is discussed to be the Rho GTPase most important for cell–cell and cell–ECM adhesion (Price and Collard, 2001) as well as for lamellipodia formation (Hall, 1998) correlates with histological grading only in case of progression from grade I to grade II, but not to grade III. One possible interpretation of this finding is that Rac function might be important only during early steps in tumour progression, but not in later steps. It is also possible that Rac activity is modulated in tumours by changes in the activity of regulatory factors such as guanine exchange factors (GEFs). In this case, Rac protein levels would remain unchanged. Therefore, future studies addressing the question of expression level and activity of Rho-regulatory factors in breast tumours are required. A correlation between RhoA-like protein expression and the amount of the HER-2/neu oncogene or p53 mutation was not observed. The same is true for RhoA expression and hormone status of the tumours (data not shown). Summarising the data, a good correlation with clinically established tumour markers was observed with RhoA-like proteins but not with Rac1 and Cdc42. Although the amount of Rho proteins in tumours was not related to their mRNA levels, we analysed whether mRNA expression of particular *rho* mRNA species might be independently related to the histological grade of breast tumours. This correlation analysis was performed since (1) about 30% of the tumours investigated showed enhanced expression of *rhoB* and *rhoC* mRNA and (2) in a recent report it has been shown that *rhoC* mRNA expression is related to malignancy of pancreas carcinomas (Suwa *et al*, 1998b). Unfortunately, studies on the level of protein expression were not included in this report. Therefore the question remains whether or not the reported increase in *rhoC* mRNA is accompanied by elevation in the amount of Rho protein. For breast tumours we found that there was only a tendency in the expression of *rhoB*, *rhoC* and *rac1* mRNA, but not of *rhoA* mRNA, to increase with grading. The tendency was most obvious for *rhoC*. However, because of the tremendous variability of *rhoC* mRNA expression in grade III tumours, a statistically significant correlation as found on the level of the protein expression was not observed. Thus, in contrast to pancreas carcinomas, *rhoC* mRNA expression level seems not to be an indicator of malignancy in breast tumours.

Since Ras is often either overexpressed or mutationally activated in a variety of human tumours, we wished to know whether the Ras-homologous GTPases are mutationally altered in breast tumours. Sequence analyses of the RhoA-like GTPases RhoA, RhoB and RhoC as well as of Rac1 failed to detect any mutations within the GTP binding or the effector binding domains of these Rho GTPases. Obviously, tumour associated changes in Rho-regulated functions are independent of mutational changes of Rho proteins itself. Rather, overexpression seems to be the predominant trait of activation of Rho signalling in breast tumours. Whether or not alterations in the activity of Rho-regulatory factors or Rho effector proteins such as Rho-kinase (ROK) or p21-associated kinases (PAK) do also occur in breast tumours will be subject of forthcoming studies.

In summary, we demonstrated that different types of Rho GTPases are highly overexpressed on protein but not on mRNA levels in breast tumours as compared to the normal tissue of the same individual. The data show at the first time that the amount of RhoA-like proteins but not of Rac1 and Cdc42, is related to clinically established prognostic breast tumour markers such as histological grade and proliferation index. A significant correlation between the expression of RhoA-like GTPases and histological grade was only observed on the protein, but not on the mRNA level. Overall, the data support the view that RhoA-like proteins are important factors involved in the development and progression of breast tumours and are of prognostic value. Moreover, the data may provide a platform for the development of novel tumour therapeutic strategies which are based on the inhibition of RhoA-like GTPases.
